# Evidence that the domesticated fungus *Leucoagaricus gongylophorus* recycles its cytoplasmic contents as nutritional rewards to feed its leafcutter ant farmers

**DOI:** 10.1186/s43008-023-00126-5

**Published:** 2023-09-15

**Authors:** Caio Ambrosio Leal-Dutra, Lok Man Yuen, Bruno Augusto Maciel Guedes, Marta Contreras-Serrano, Pedro Elias Marques, Jonathan Zvi Shik

**Affiliations:** 1 Section for Ecology and Evolution, Department of Biology, University of Copenhagen, Universitetsparken 15, 2100 Copenhagen, Denmark; 2 Present Address: Department of Biology, ETH Zürich, Universitätsstrasse 16, Zürich, 8092 Switzerland; 3 Laboratory of Molecular Immunology, Department of Microbiology, Immunology and Transplantation, Rega Institute, KU Leuven, Leuven, Belgium; 4 Departamento de Ciências Básicas da Vida, Universidade Federal de Juiz de Fora, Campus Governador Valadares, Governador Valadares, MG 35020-360 Brazil; 5 Smithsonian Tropical Research Institute, Apartado 0843-03092 Balboa, Ancon, Republic of Panama

**Keywords:** Autophagy, *Leucoagaricus gongylophorus*, Leafcutter ant, Gongylidia, Fungus, Symbiosis

## Abstract

Leafcutter ants farm a fungal cultivar (*Leucoagaricus gongylophorus*) that converts inedible vegetation into food that sustains colonies with up to millions of workers. Analogous to edible fruits of crops domesticated by humans, *L. gongylophorus* has evolved specialized nutritional rewards—swollen hyphal cells called gongylidia that package metabolites and are consumed by ant farmers. Yet, little is known about how gongylidia form, and thus how fungal physiology and ant provisioning collectively govern farming performance. We explored the process of gongylidium formation using advanced microscopy to image the cultivar at scales of nanometers, and both in vitro experiments and in silico analyses to examine the mechanisms of gongylidia formation when isolated from ant farmers. We first used transmission electron, fluorescence, and confocal microscopy imaging to see inside hyphal cells. This imaging showed that the cultivar uses a process called autophagy to recycle its own cellular material (*e.g*. cytosol, mitochondria) and then shuttles the resulting metabolites into a vacuole whose continual expansion displaces other organelles and causes the gongylidium cell’s bulging bulb-like appearance. We next used scanning electron microscopy and light microscopy to link this intracellular rearrangement to the external branching patterns of gongylidium cells as they clump together into edible bundles called staphyla. We next confirmed that autophagy plays a critical role in gongylidium formation both: (1) in vitro as gongylidium suppression occurred when isolated fungal cultures were grown on media with autophagy inhibitors, and (2) in silico as differential transcript expression (RNA-seq) analyses showed upregulation of multiple autophagy gene isoforms in gongylidia relative to undifferentiated hyphae. While autophagy is a ubiquitous and often highly derived process across the tree of life, our study reveals a new role for autophagy as a mechanism of functional integration between ant farmers and their fungal crop, and potentially as a signifier of higher-level homeostasis between uniquely life-time committed ectosymbionts.

**Supplementary Information:**

The online version contains supplementary material available at 10.1186/s43008-023-00126-5.

## INTRODUCTION

The advent of domesticated agriculture some 10,000 years ago was a turning point for humans and for the domesticated crops whose derived traits would likely have been maladaptive in their free-living ancestors (Milla et al. 2015; Gering et al. 2019; Solberg et al. 2020). Key crop adaptations include whole genome duplication events (resulting in polyploidy) that can increase functional heterozygosity (Comai 2005) and selection for specific regulatory genes that can reduce seed shattering or enhance fruit size, color, and sweetness (Piperno 2011; Stetter et al. 2017; Edger et al. 2019; Renner et al. 2021). Fascinatingly, humans are not the only farmers. Several insect lineages independently evolved obligate farming systems of fungal cultivars that produce specialized edible reward structures (Mueller et al. 2005). However, while human farmers modify growth environments in well-known ways to maximize crop yield (e.g. adding fertilizers, controlling watering conditions, etc.), the analogous mechanisms by which insect farmers promote expression of edible reward structures in fungal cultivars remain poorly understood.

The largest-scale insect farmers are the *Atta* leafcutter ants, the crown group of the fungus-farming ‘attine’ ant lineage (Schultz and Brady 2008; Barrera et al. 2021). A mature rainforest colony of the leafcutter ant *Atta colombica* can have millions of specialized ants that divide the work of foraging fresh plant fragments and caring for the fungal cultivar (Hölldobler and Wilson 2011). In this way, colonies convert foraged fragments from hundreds of plant species (Wirth et al. 2003) into a mulch used to provision their fungal cultivar *Leucoagaricus gongylophorus*. In return, the cultivar converts inedible plant biomass into edible reward structures called gongylidia, which are swollen hyphal cells *ca.* 30 µm in diameter that grow in bundles called staphylae (Möller 1893; Swingle 1896; Wheeler 1907; Weber 1957, 1966; Quinlan and Cherrett 1979; Powell 1984; Mueller et al. 2001; Crumière et al. 2021). Gongylidia are a defining trait of crop domestication and are unique to the fungal lineage farmed by the *Atta* and *Acromyrmex* leafcutter ants and other higher-neoattine genera including *Trachymyrmex*, *Sericomyrmex*, *Mycetomoellerius*, and *Paratrachymyrmex* (Mueller et al. 2001; Nygaard et al. 2016; Solomon et al. 2019).

Fungal gongylidia mediate functional integration with their ant symbionts in two main ways. First, they contain enzymes (*e.g*., laccases, pectinases, proteases) that ants ingest and then vector to patches of newly deposited vegetation to catalyze fungus-mediated digestion and detoxification (Schiøtt et al. 2010; De Fine Licht et al. 2013; Kooij et al. 2014; Aylward et al. 2015; Schiøtt and Boomsma 2021). Second, they contain nutrients (*e.g.*, amino acids, lipids, and glycogen) that are the ants’ primary food source (Martin 1970; Martin and Martin 1970). Despite their importance to farming system success, surprisingly little has been discovered about gongylidium structure, morphology, or development in the leafcutter cultivar since the work of Angeli-Papa and Eymé (1979, 1985) performed nearly 40 years ago. These researchers used transmission electron microscopy (TEM) imaging to observe endoplasmic reticulum membranes engulfing mitochondria in developing gongylidia and infer that a cellular degradation process called autophagy was involved in gongylidium formation (Angeli-Papa and Eymé, 1979). This is consistent with subsequent research showing that while the metabolic pathways mediating autophagy are conserved across fungi, it has evolved diverse functions across fungi including cellular development (Pinan-Lucarré et al. 2003; Kües and Navarro-González 2015), differentiation (Kikuma et al. 2006; Liu et al. 2012) and housekeeping processes (Bartoszewska and Kiel 2011; Elander et al. 2017). Despite major advances in the field, the initial work of Angeli-Papa and Eymé has, to our knowledge, not been validated using experimental and/or modern -omics approaches, and the implications of such cultivar altruism have not been examined using the lens of symbiotic co-evolution.

Subsequent indirect evidence for autophagy came from Bass and Cherrett (1996) who showed that staphyla density increases when leafcutter workers were allowed to ‘prune’ their cultivar. This is because pruning likely severs small bundles of hyphae from the delivery networks that supply metabolites derived from ant-provisioned plant fragments. We hypothesize that this result is relevant to the prediction of an autophagic mechanism since starvation often induces autophagy within cells (Noda and Ohsumi 1998). Specifically, a cell undergoing autophagy incorporates its own cytoplasmic components (*i.e.*, glycogen, proteins, organelles) into vacuoles where their enzymatic degradation yields catabolites that are recycled as nutrients to sustain other cellular processes (Levine and Klionsky 2004; Klionsky et al. 2021). Here, we explicitly tested the hypothesis that autophagy and plant-fragment provisioning by ants provide alternative and/or complementary pathways for gongylidium formation.

We further predicted that the existence of an autophagic gongylidium production pathway would be important for mediating symbiotic stability. On one hand, ant farmers benefit by being able to regulate gongylidia quantity and quality through their own prudent plant-fragment provisioning decisions. Indeed, many decades of research have highlighted the unique adaptations of leafcutter ants for selecting, cutting, and carrying suitable plant fragments for cultivar provisioning (Hölldobler and Wilson 2011). On the other hand, cultivar-mediated autophagy would imply that natural selection has targeted the farming symbiosis in ways that made provisioning more robust and less dependent on the variable quality and quantity of foraged vegetation. Specifically, autophagic nutrient recycling of cellular contents would: 1) reduce variability in the quality of the cultivar’s nutritional rewards and provide the cultivar with opportunities to fine-tune the composition of its metabolic source material, 2) constrain the ability of ants to directly regulate cultivar productivity through their provisioning decisions, and 3) function in a complementary manner to provisioned plant substrates by providing a stable supply of metabolic precursor substrates during periods of environmental vegetation shortage.

Here, we described the stages of gongylidium formation and determined the importance of autophagy in this process by integrating advanced imaging, experimental, and -omics approaches. First, we visualized the morphology of gongylidia and staphylae using scanning electron microscopy and then described the cellular reorganizations that mediate gongylidium formation using light, fluorescence, and transmission electron microscopy. Second, we tested whether autophagic pathways are differentially expressed in developing gongylidium cells by performing transcriptomic analyses of the undifferentiated mycelia and staphylae of the cultivar when grown under controlled in vitro conditions on a standardized medium without ant farmers. Third, we tested whether autophagy is necessary for gongylidium formation by performing an in vitro experiment where the density of staphyla was measured in cultivars grown with known inhibitors and promoters of autophagy in fungal cells.

## METHODS

### Sample acquisition and fungal symbiont isolation

The two colonies of *Atta colombica* (Ac2012-1 and Ac2019-1) used in this study were collected in Soberanía National Park, Panama and thereafter maintained at the University of Copenhagen in a climate-controlled room (25 °C, 70% RH, minimal daylight). For microscopy and imaging, we used staphylae collected directly from the colonies’ fungus gardens, and for the autophagy inhibition experiments and transcriptome sequencing, we used axenic cultures isolated from the fungal cultivar (*L. gongylophorus*) grown in 90 mm Petri dishes filled with 20 ml potato dextrose agar (PDA) that were kept in the dark at 25 °C. Staphylae naturally include many individual gongylidia comprising a broad range of developmental stages (and bulb sizes). While we did not determine the age of any individual gongylidium cell, the images we acquired capture this natural variation.

### Imaging morphology of staphyla and gongylidia

We used scanning electron microscopy (SEM) to visualize the external morphology of gongylidia and staphylae. The samples were fixed in PBS with 0.1% Tween 20 and fixatives (4% glutaraldehyde, 4% formaldehyde) and then dehydrated in an ethanol series (35%, 55%, 75%, 85%, 95%, and 2 × in 100%) for 30 min per concentration, critical-point dried, coated with platinum, and imaged on a JSM-840 scanning electron microscope (JEOL, Tokyo, Japan) at 7.0 kV at the Zoological Museum of the University of Copenhagen. A slight wrinkled appearance of the surface of gongylidium cells in the resulting SEM images was due to unavoidable plasmolysis caused by the preparation process.

We then used light, fluorescence and confocal microscopy to view the cultivar’s internal morphology (*e.g*. septa, vacuoles, nuclei, etc.) and examine the cellular reorganizations associated with gongylidium formation. For all imaging approaches, staphylae were first placed in a drop of mounting solution (dH_2_O, 3% KOH or phosphate-buffered saline) on a glass slide. Gongylidia were then separated under a stereo microscope (16 × or 25 × magnification) with 0.16-mm diameter acupuncture needles and stained. For visualization with white light, we placed samples in either 0.1% Congo-red (in 150 mM NaCl) for one minute followed by a wash with 150 mM NaCl, or 1.5% phloxine B followed by a wash with 3% KOH. For nucleus visualization under UV light, we stained samples for 10 min using “Vectashield with DAPI” (Vector Laboratories, Burlingame, CA, USA). We then acquired images at magnifications of up to 400 × by performing bright-field, dark-field, phase-contrast, and fluorescence microscopy under an Olympus BX63 microscope (Olympus, Tokyo, Japan). The microstructures were measured and photographed using a mounted QImaging Retiga 6000 monochrome camera and cellSens Dimension v1.18 (Olympus) image-processing software. For confocal imaging, we stained the staphylae with dextran conjugated with Alexa Fluor 647 (Invitrogen, MA, USA) in the concentration of 100 µg/ml in PBS for 30 min and washed twice in PBS. Confocal images were acquired with a Dragonfly spinning-disk confocal system (Andor Technology, Belfast, Northern Ireland) equipped with a 25 × water-immersion objective. Samples were excited with a 637 nm laser line and fluorescence was collected with a 698/77 emission filter. Images were processed using FIJI software.

We next used transmission electron microscopy (TEM) to visualize fungal cells with greater magnification and resolution (i.e. the 500 nm scale). Staphylae were collected from fragments of intact fungus gardens, fixed in 2% glutaraldehyde in 0.05 M PBS (pH 7.2) and then post-fixed in 1% w/v OsO_4_ with 0.05 M K_3_Fe(Cn)_6_ in 0.12 M sodium phosphate buffer (pH 7.2) for 2 h at room temperature. Fixed samples were washed three times in ddH_2_O for 10 min and dehydrated in a series of increasing ethanol concentrations (70%, 96% and 99.9%) 2 × 15 min per concentration. Samples were then repeatedly infiltrated for 20 to 40 min with increasing Resin Epon:Propylene oxide ratios (1:3, 1:1, 3:1) and subsequently embedded in 100% Epon and polymerized overnight at 60 °C. Sections of 60 nm thickness were then cut with an Ultracut 7 ultramicrotome (Leica, Vienna, Austria), collected on copper grids with Formvar supporting membranes, and stained with both uranyl acetate and lead citrate. These samples were TEM imaged on a CM100 BioTWIN (Philips; Eindhoven, The Netherlands) at an accelerating voltage of 80 kV. Digital images were recorded with a side-mounted OSIS Veleta digital slow scan 2 × 2 k CCD camera and the ITEM software package (Olympus Soft Imaging Corp, Münster, Germany). This sample preparation and imaging was performed at the Core Facility for Integrated Microscopy at the University of Copenhagen.

### Autophagy inhibition assay

We tested whether autophagy is necessary for gongylidium formation using an in vitro growth assay with four treatment groups. Autophagy is often initiated in cells when the target of rapamycin kinase (TOR) is inhibited and can thus be induced in vitro by adding rapamycin (RAP) (Noda and Ohsumi 1998), an allosteric TOR inhibitor (Klionsky et al. 2021). Autophagy is often inhibited in vitro using Chloroquine (CQ) or 3-methyladenine (3-MA) as these compounds respectively block autophagosome-vacuole fusion (Mauthe et al. 2018) and suppress an enzyme (class III PtdIns3K) required to initiate autophagosome formation (Wu et al. 2010). We compared cultivars grown in the dark for 46 days at 25 °C on a baseline PDA medium containing nutrients known to maximize cultivar performance (Crumière et al. 2021; Shik et al. 2021) (n = 40) with cultivars grown on plates with RAP (n = 27), CQ (n = 28), or 3-MA (n = 30). Briefly, 5-mm diameter fungus plugs from previously isolated and reinoculated PDA culture were placed in 60-mm Petri dishes containing 10 ml of PDA (control), PDA + 300 ng/ml rapamycin (Medchem Express, Monmouth Junction, NJ, USA), PDA + 1.5 mM chloroquine diphosphate (Sigma-Aldrich, St. Louis, Missouri, USA), or PDA + 10 mM 3-MA (Medchem Express). We then photographed plates to measure growth area (mm^2^) using ImageJ (Schneider et al. 2012) and directly counted the staphylae on these Petri dishes under a stereo microscope (40 × magnification) to quantify staphyla density (number of staphylae/growth area). The measurement of growth area also enabled assessment of other unintended inhibitory effects of the added chemicals on cultivar performance. We tested for treatment effects (PDA-Control, RAP, CQ, 3-MA) on mycelial growth and staphyla density in R version 4.0.2 (R Core Team 2020) using a Kruskal–Wallis test in *rstatix* version 0.7.0 (Kassambara 2021) with pairwise post-hoc tests performed using Dunn’s test in *rstatix* with adjusted p values calculated using the false-discovery rate method.

### Transcriptome sequencing, assembly, and differential expression analyses

To detect whether upregulated transcripts in staphylae were enriched with autophagy genes, we first collected staphylae and undifferentiated mycelia from axenic fungal cultures isolated from an *A. colombica* colony (Ac2012-1) and grown on PDA for 30 days as specified above. From individual Petri dishes, we then used a RNeasy plant mini kit (Qiagen, Germany) to extract total RNA from differentiated staphyla (pooled 200 staphylae individually collected with sterile acupuncture needles, n = 5 Petri dishes samples) and undifferentiated mycelia (adjacent mycelia lacking staphylae, n = 5 Petri dishes samples). Samples were immediately placed in Qiagen RLC buffer containing 40 mM dithiothreitol (DTT) and RNA was then extracted using the manufacturer-specified protocol. These samples were sent to BGI Europe (Copenhagen, Denmark) where libraries were constructed with mRNA enrichment with oligo-dT primers. For each sample, between 24 and 30 million clean 100 bp paired-end reads were generated by a DNBseq-G400 sequencer (MGI Tech, Shenzhen, China).

We used pooled clean reads from all samples (staphylae and mycelia) to assemble a de novo transcriptome using Trinity-v2.12.0 (Grabherr et al. 2011) and default settings with the addition of Jaccard-clip option, aimed at reducing the generation of chimeric transcripts. Using CD-HIT (Fu et al. 2012) to cluster highly similar sequences (98% similarity), we reduced the assembly from 66,224 to 62,102 transcripts ranging from 186 to 22,674 bp. We used Trinity built-in pipelines to first estimate transcript abundance with RSEM v1.3.1 (Li and Dewey 2011) and then build a transcript expression matrix. We then performed a differential expression analysis with another trinity built-in pipeline using DESeq2 (Love et al. 2014) setting the analysis to filter for the differentially expressed transcripts (DETs) with log_2_ fold change > 2.0 and p-value < 0.001 (Benjamini–Hochberg method corrected Wald test). To annotate the DETs, we first converted transcripts to amino acid sequences using Transdecoder (https://github.com/TransDecoder/TransDecoder) to predict and retain only the single best open read frame per transcript and translate them into amino acid sequences, and then annotated the DETs with KEGG database (Kanehisa and Goto 2000) using the online tools BlastKOALA, GhostKOALA (Kanehisa et al. 2016) and KofamKOALA (Aramaki et al. 2019). In addition, we also run Proteinortho v6.0.11 (Lechner et al. 2011) to identify yeast autophagy orthologous genes. Proteinortho was conducted twice searching for orthologues with diamond v.0.8.22.84 (Buchfink et al. 2015) and blastp v.2.12.0 + (Camacho et al. 2009). Orthologs found in these analyses were merged and summarized in Table 1 and Additional file 4: Fig. S3.

**Table 1:** Differentially expressed transcripts in the autophagy pathway annotated with KEGG database (see Additional file 4: Fig. S3)

Tissue	KEGG	Gene name	No. of transcripts
Staphylae	K17888	ATG10; Ubiquitin-like-conjugating enzyme ATG10L	1
	K06902	ATG22; MFS transporter, UMF1 family	1
	K17900	ATG15; Lipase ATG15	2
	K17260	ARP2; Actin-related protein 2	1
	K04382	PPH21; Serine/threonine-protein phosphatase 2A catalytic subunit	1
	K12761	SNF1; Carbon catabolite-derepressing protein kinase	1
	K01336	PRB1; Cerevisin	4
	K20195	MON1; Vacuolar fusion protein MON1	1
	K04345	PKA; Protein kinase A	1
	K01381	PEP4; Saccharopepsin	1
	K07897	YPT7; Ras-related protein Rab-7A	1
	K08334	ATG6; Beclin	1
	K08337	ATG7; Ubiquitin-like modifier-activating enzyme ATG7	1
	K16196	GCN2; Etranslation initiation factor 2-alpha kinase 4	1
	K20177	VPS3; Vacuolar protein sorting-associated protein 3	7
Mycelium	K06902	ATG22; MFS transporter, UMF1 family	1
	K00654	LCB2; Serine palmitoyltransferase	1
	K01336	PRB1; Cerevisin	1
	K07827	RAS; GTPase KRas	1
	K12767	RIM15; Serine/threonine-protein kinase RIM15	1
	K19800	SCH9; Serine/threonine protein kinase SCH9	1
	K20183	VSP39; Vacuolar protein sorting-associated protein 39	1
	K01381	PEP4; Saccharopepsin	1

Due to alternative splicing, several transcripts derived from the same gene or containing the same domain are present, leading to transcripts from the same gene/domain being up- or downregulated

## RESULTS

### Gongylidia as nutritional reward traits

**Fig. 1:**
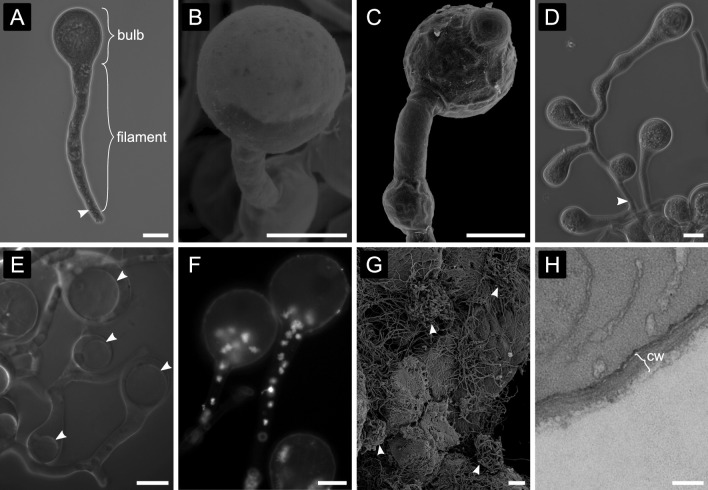
The *Leucoagaricus gongylophorus* fungal cultivar produces gongylidia as specialized nutritional reward structures to feed leafcutter ants. **A**–**C** Gongylidium cells are typically depicted as a bulb at the end of a filament in the apical hyphal compartment separated by a septum (arrowheads). **D** Gongylidia frequently exhibit more complex branching patterns, with bulbs between filaments or in lateral branches of single hyphal cells delimited by septa. **E** Each gongylidium cell contains a single large vacuole (arrowheads). **F** Individual gongylidium cells are polykaryotic (Kooij et al. 2015), meaning that they have many haploid nuclei (white dots visualized using DAPI staining). Here, we show that in mature non-branching gongylidium cells, these nuclei occur at the base of the bulb (below a single large vacuole) and in the filament. **G** Staphylae grow in discrete patches at the surface of the fungus garden matrix in the middle garden stratum (arrowheads). **H** Gongylidium cells have thin cell walls ranging from 120 to 220 nm (**cw**). Images produced by light microscopy (panels **A**, **D**, **F**), fluorescence microscopy stained with DAPI (panel **E**), SEM (panels **B**, **C**, **G**) and TEM (panel **H**). Scale bars: A-**F** = 20 μm, **G** = 100 μm, **H** = 200 nm

Each gongylidium cell consists of two sections that we term the bulb (swollen section) and the filament (elongated section) (Fig. 1 A). Gongylidia are often connected by intercalary bulbs (between filaments) and intercalary filaments (between bulbs) (Fig. 1 B-C) with multifaceted branching patterns and individual gongylidium cells bearing two or more terminal bulbs (Fig. 1 D). Gongylidium bulbs usually contained one vacuole (Fig. 1 E) that comprised up to half of each bulb’s total volume. Gongylidia had variable bulb diameters ranging from 12 µm to 50 µm and variable filament lengths ranging from 40 µm to > 250 µm, possibly due to their developmental stage. Our observations further suggest that if they are uneaten, these bulbs expand indefinitely as old staphylae isolated from ants in agar culture eventually burst and leave a liquid droplet. Variable bulb sizes may reflect these indeterminate growth trajectories.

We also confirmed a previous observation that *L. gongylophorus* is polykaryotic (Kooij et al. 2015) as all gongylidium cells in our study also contained at least eight nuclei. However, our imaging further suggests that gongylidium branching morphology is mediated by intracellular nucleus distributions. Specifically, nuclei in mature gongylidium cells are usually concentrated at the intersection of the bulb and the filament (Fig. 1 F).

Individual staphylae range widely in size, being composed of tens to hundreds of individual gongylidium cells (Fig. 1 G, Additional file 1: Fig. S1). Staphylae were always formed on the surface of fungus garden mycelial matrix where ants can readily detach them from surrounding hyphae. Staphylae also form in the absence of ants under in vitro (Petri dish) growth conditions, but they have the following key morphological differences compared to those growing in ant-tended fungus gardens, being: 1) less detachable because they are usually covered by an overgrowth of filamentous hyphae, 2) larger in area and with more individual gongylidium cells, and 3) covered to some extent with fluids from ‘burst’ gongylidium cells. It is possible that under farming conditions, ants harvest staphylae earlier in their development before vacuoles can produce turgor pressure that exceeds the retaining capacity of their exceptionally thin (ca. 120 to 220 nm) cell walls (Fig. 1 H).

### Visual evidence for an autophagic mechanism of gongylidium formation

**Fig. 2:**
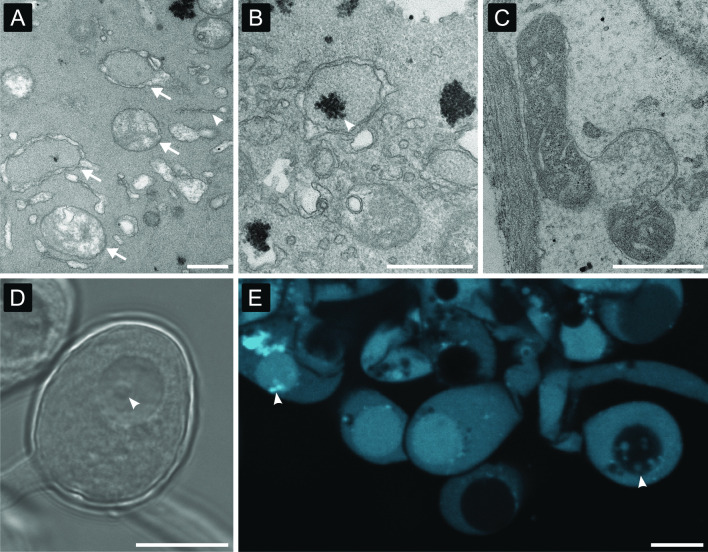
Autophagic recycling of cellular material. **A** The autophagosomes that are diagnostic of autophagy are vesicles with double-layered membranes (arrows) and are produced by stretches of endoplasmic reticulum membranes (arrowheads). **B** Autophagosomes sequester cytoplasmic components including glycogen (arrowhead) and **C** mitochondria that are then delivered to vacuoles in gongylidium bulbs for further degradation. **D**–**E** Vacuolar expansion is mediated by fusion of autophagosomes that lose their outer membrane once they fuse with the vacuole. These single-membraned autophagic bodies are vesicles that can be seen inside the vacuole prior to their degradation (arrowhead). Images acquired by TEM (**A**–**C**), phase contrast microscopy (**D**) and confocal microscopy stained with dextran-Alexa Fluor 647 (**E**). Scale bars **A**–**C** = 500 nm, **D**–**E** = 20 μm

Microscopy images (TEM, light, fluorescence and confocal) of gongylidium cells revealed structures that are diagnostic of macroautophagic processes. First, gongylidia were enriched with long stretches of endoplasmic reticulum that produce double-membraned vesicles called autophagosomes (Fig. 2), within which the recycling of cellular materials is initiated. We confirm that autophagosomes contained cytosol (Fig. 2 A), glycogen (Fig. 2 B) and mitochondria (Fig. 2 C), and predict that other metabolites (*e.g*., lipids, amino acids, enzymes (De Fine Licht et al. 2013; Grell et al. 2013; De Fine Licht et al. 2014; Nygaard et al. 2016; Khadempour et al. 2021)) that are too small to be detected with TEM are also abundant. Second, large numbers of damaged mitochondria were present in gongylidia and were often associated with endoplasmic reticulum membranes (Fig. 2 C) where they were likely destined to be sequestered into autophagosomes, digested, and recycled into edible metabolites. Third, vacuoles within gongylidium bulbs often contained single-membraned autophagic bodies (Fig. 2 D-E). These vesicles indicate the delivery of metabolites into vacuoles, since they are autophagosomes that lost their outer membrane after vacuolar fusion. This was confirmed by confocal images showing that autophagic bodies within gongylidium vacuoles contained fluorescently labeled sugars from the cultivar’s cytosol (Fig. 2E, Additional file 2: Video S1).

This combined visual evidence does not support the hypothesis that gongylidia promote vacuolar expansion by ingesting substances from the environment (i.e., endocytosis). First, endocytosis produces single-membrane vesicles (derived from plasmalemma invagination) that were not observed in the cytoplasm, and these vesicles would not generate autophagic bodies when fused to the vacuole. Second, since gongylidia are not formed in contact with substrate (Powell 1984), they are not able to endocytose nutrients from the environment.

### Experimental in vitro evidence for an autophagic mechanism

**Fig. 3:**
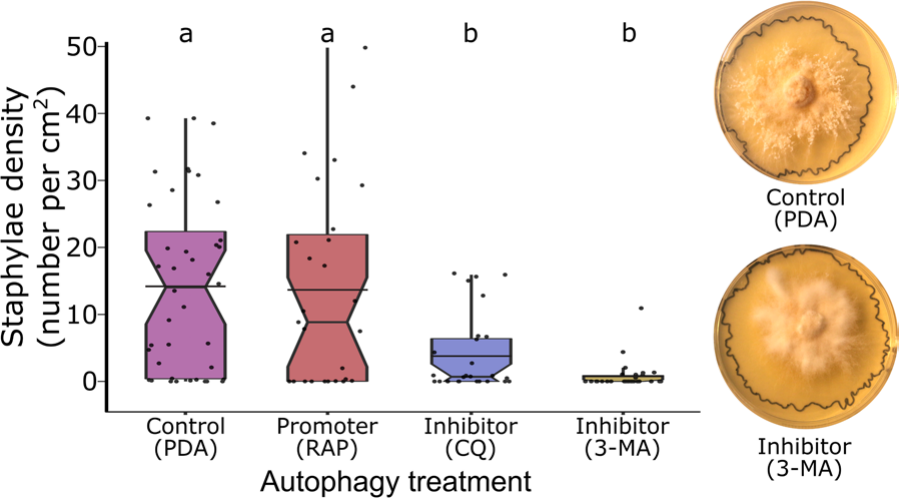
Experimental evidence that autophagic recycling of the fungal cultivar’s own cellular material mediates gongylidium formation. Gongylidium density was significantly inhibited when L. gongylophorus was grown on potato dextrose agar supplemented with either autophagy inhibitor, chloroquine (CQ, n = 28) or 3-methyladenine (3-MA, n = 30), relative to control (PDA, n = 40) and an autophagy promoter rapamycin (RAP, n = 27). Representative Petri dishes with control (PDA; top) and inhibition (CQ; bottom) are displayed at the right; black outlines indicate the radial growth area of cultivars and white fungal clusters in the control are the staphylae. Different letters above the boxes indicate significant differences determined by a post-hoc Dunn’s pairwise test (*p* < 0.05) and horizontal bars indicate the distribution means

An autophagic mechanism for gongylidium formation was further supported by significant in vitro treatment effects of chemical autophagy inhibitors on staphyla density (Kruskal–Wallis: H_3_ = 34.7, *p* < 0.001, Fig. 3). Pairwise post-hoc comparisons indicated that both autophagy inhibitors (3-MA and CQ) significantly reduced staphyla density compared to both the control (PDA) (*p*_*adj*_ < 0.001 and *p*_*adj*_ = 0.001, respectively) and the autophagy-promoter (RAP) treatment (*p*_*adj*_ < 0.001 and *p*_*adj*_ = 0.026, respectively). There was also a significant treatment effect on mycelial growth area (H_3_ = 16.3, p = 0.001) that was due to differences between 3-MA and all other treatments (PDA:3-MA, *p*_*adj*_ = 0.004; RAP:3-MA, *p*_*adj*_ = 0.015; CQ:3-MA, *p*_*adj*_ = 0.001), with no other significant pairwise comparisons (Additional file 3: Fig. S2). Thus, while both autophagy inhibition treatments resulted in staphyla reduction, it is possible that 3-MA negatively influenced staphyla density through unknown indirect effects on cultivar performance. The autophagy promotor (RAP) did not significantly increase staphyla density relative to control (PDA) or either of the autophagy inhibition treatments (Fig. 3).

### Transcriptomic evidence for an upregulated autophagic pathway in gongylidium cells

Using a *de-novo* assembly and a clustering analysis by similarity, we recovered 62,102 transcripts from the *L. gongylophorus* transcriptome. Of these, 2,523 were differentially expressed (log2 fold-change > 2, Benjamini–Hochberg adjusted *P* < 0.001) in staphylae (n = 1,765 transcripts) or in non-differentiated mycelia (n = 758 transcripts). Of these differentially expressed transcripts (DETs), 33 were assigned a KEGG orthology term associated with the yeast autophagy pathway (n = 25 in staphylae, n = 8 in mycelia, Table 1) representing 20 genes from the pathway. Several key genes that were upregulated in staphylae typically have elevated transcription during autophagy (Klionsky et al. 2021). These include genes linked to the recruitment of cargo into incipient unclosed membranes (*i.e.*, phagophores) and mature autophagosomes (ATG7 and ATG10), as well as genes related to starvation signaling (PKA, SNF1, PPH21, GCN2, ARP2 and ATG6), vacuole fusion machinery (YPT7, MON1 and VPS3), degradation of autophagic bodies (PEP4, PRB1 and ATG15) (Table 1, Additional file 4: Fig. S3) and efflux of recycled material (ATG22). Three genes were represented by upregulated isoforms in both mycelium and staphylae (PEP4, PRB1 and ATG22).

## DISCUSSION

Our results suggest that the fungal cultivar can mediate higher-level homeostasis of leafcutter farming systems in a manner potentially decoupled from provisioning decisions of ant farmers. Several lines of evidence indicate that the fungal cultivar uses autophagic recycling to convert its own cellular material into edible metabolites and promote the expansion of its nutritional reward structures by filling them with these metabolites. First, nanoscale advanced imaging shows the cellular hallmarks of autophagy (*e.g*. autophagosomes, autophagic bodies, abundant endoplasmic reticula) and indicates rapid delivery within 30 min of labeled cytosol nutrients into gongylidium vacuoles. Second, we provide experimental evidence that this cellular recycling process is necessary for maximal production of nutritional reward structures, since gongylidia density was reduced following experimental suppression of autophagy. Third, we show that autophagy gene expression pathways are upregulated in gongylidium cells. Based on this evidence, we hypothesize that this autophagic recycling pathway represents a final domestication step where the cultivar came to unambiguously prioritize nutritional services to its hosts even at the expense (up to a point) of its own mycelial health.

This contention is not meant to discount the importance of plant fragment provisioning by ants for optimized cultivar production. For instance, evidence suggests that the density of staphyla can be mediated by the match between nutrients used to provision the fungal cultivar and its fundamental niche requirements for these nutrients (Shik et al. 2021). Moreover, ample evidence suggests the cultivar directly metabolizes provisioned nutrients and shunts edible metabolites into gongylidia. First, the cultivar can metabolize lipids rich in alpha-linoleic acid (18:3) from provisioned plant fragments into linolenic acid (18:2) that is enriched in gongylidia (Khadempour et al. 2021) and can elicit attractant behaviors in ant workers (Khadempour et al. 2021). Second, isotopic enrichment studies have shown that the cultivar quickly (within two days) shunts C and N from provisioned substrates (glucose and ammonium nitrate, respectively) into edible gongylidia (Shik et al. 2018). Yet, while much research assumes that cultivar production hinges on optimized foraging by ants, it is possible that targeted nutritional suppression mediated by ant pruning behaviors (Bass and Cherrett 1996) are actually important triggers of autophagic processes and thus farming system productivity.

In this sense, the autophagic recycling pathway can be seen as a trait that could evolve in an obligately symmetric commitment between symbionts achieved after the domesticated fungal cultivar lost the capacity for a free-living existence and became fully integrated into the host colony’s germ line. Consider that, despite clear analogies with farming systems of humans (Mueller et al. 2005), farming by leafcutter ants is fundamentally different because it is ‘organismal’ in the sense that it represents a strictly symmetrical obligate mutualistic dependence (Boomsma 2022). Such an arrangement usually does not allow for alternative crops, but it does sustain selection for co-evolutionary integration and higher-level adaptation that cannot evolve when farming practices are asymmetrically promiscuous (Axelrod and Hamilton 1981; Frank 1996). We propose that autophagic recycling by the fungal cultivar for the purpose of provisioning ant ectosymbionts represents an organismal level of trait evolution typically only seen in lifetime committed endosymbioses (*e.g*. the mitochondria-nucleus partnership in eukaryotic cells (Leigh Jr 2010; Boomsma and Gawne 2018)). Analogous adaptations aimed solely at regulating homeostasis at higher levels of organization are absent from other ectosymbioses that are promiscuous by comparison, where the interests of symbionts are not completely aligned, and partners must screen, sanction, and police to dissuade cheating (Heil and McKey 2003; Kiers et al. 2003; Jandér and Herre 2010).

So, how might autophagic recycling promote higher-level homeostasis? We propose that it provides a mechanism for stabilizing the quantity and fine-tuning the nutritional quality of the cultivar’s nutritional rewards in fluctuating environments. First, the seasonal and spatial availability of preferred plant fragments may vary in suboptimal ways (Wirth et al. 2003). Second, foraged plant fragments can contain key nutrients—but these nutrients can occur in suboptimal ratios and concentrations relative to the cultivar’s intrinsic needs and tolerances (Crumière et al. 2021). Third, plant fragments contain a wealth of recalcitrant compounds (e.g. cellulose and lignin) and toxic metabolites that can reduce cultivar performance (Howard 1987, 1988; Nichols-Orians and Schultz 1990; Crumière et al. 2022). During such periods where biochemically preferred plant fragments are unavailable (Berish 1986; Howard et al. 1988; Mundim et al. 2009), the cultivar may use autophagic recycling of its own organelles to yield reliably available and chemically predictable metabolic precursor compounds.

### Reconstructing the cellular reorganizations enabling gongylidium formation

**Fig. 4:**
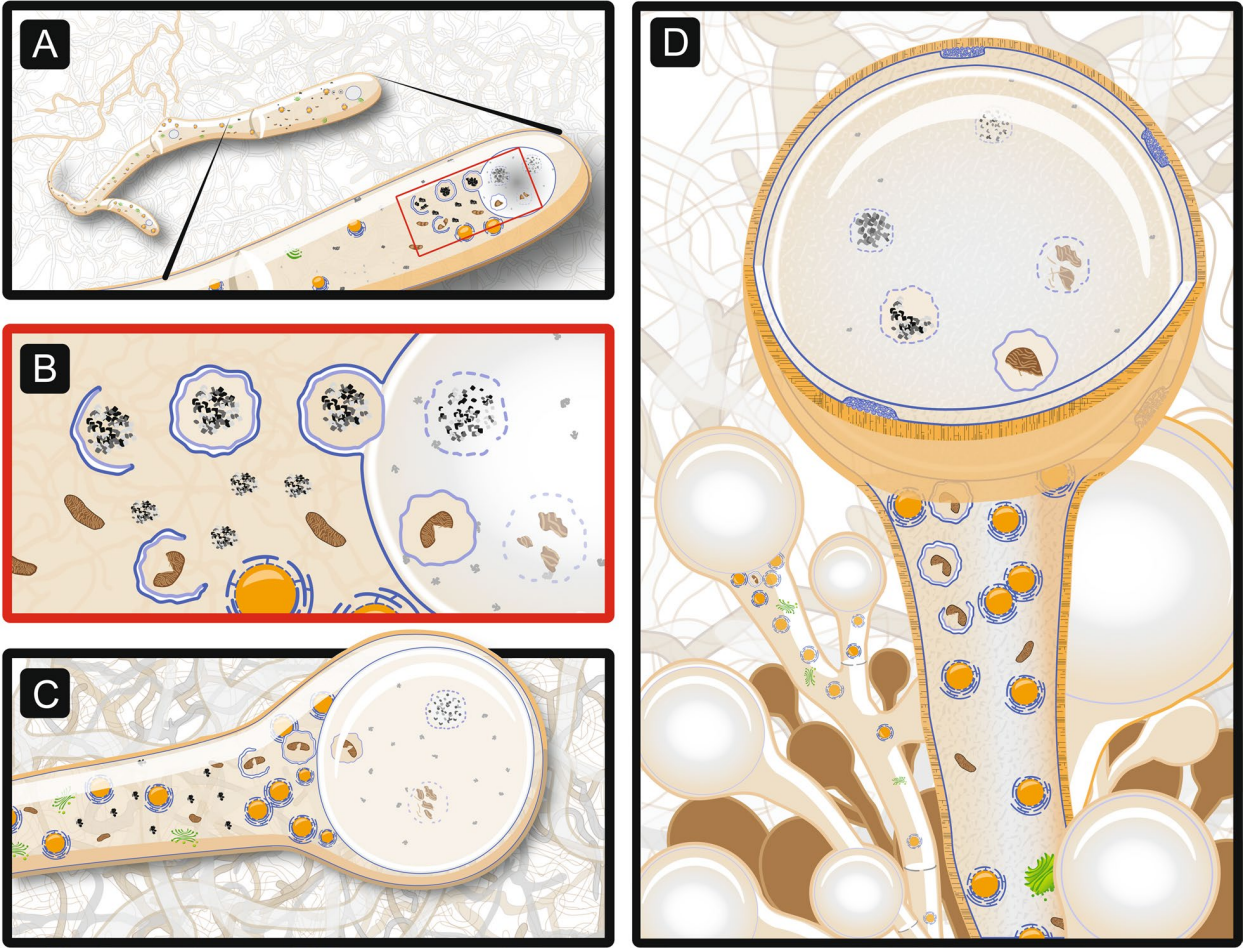
The hypothesized stages of autophagy-mediated gongylidium development. **A** An unknown mechanism (potentially starvation mediated by ant pruning (Bass and Cherrett 1996)) triggers initial hyphal widening. As a hypha elongates, its nuclei (orange circles) tend to be located near the hyphal tip. **B** Mediated primarily by an autophagic process, a large vacuole expands via the fusion of newly-formed double membrane vesicles called autophagosomes (blue membraned vesicles) that sequester material present in the cytosol like glycogen (black and gray aggregates) and damaged mitochondria (brown indented ovals). This process is indicated by the proliferation of endoplasmic reticulum membranes (blue membranes around nuclei) that produce autophagosomes. **C** The fusion of autophagosomes into vacuoles mediates apical bulb swelling and halts further apical growth by excluding nuclei from the hyphal tip. If stray nuclei remain at the hyphal tip, intercalary bulbs are formed. **D** This process repeats in up to hundreds of adjacent hyphae that become tangled to form the staphyla

The combined evidence we present throughout this study enables us to propose a complete pathway for gongylidium development (Fig. 4). The first differentiation step initiates when a hyphal cell growing at surface interstices in the fungus garden matrix widens (Fig. 4 A) and continues as a vacuole begins expanding in response to autophagic recycling. This process accelerates as autophagosomes fuse with the growing vacuole and deliver cytoplasmic cargo (Fig. 4 B) while nuclei migrate below the vacuole at the base of the expanding bulb. We hypothesize that the vacuole thus halts further apical growth by blocking communication between nuclei and the Spitzenkörper—the organized machinery for hyphal growth located in the hyphae tip (Fig. 4 C). Eventually, the indefinite vacuolar swelling forces cell wall expansion of the gongylidium bulb. Our metabolic pathway analysis revealed two upregulated isoforms of a gene related to efflux of recycled material from the vacuole (ATG22) in the staphylae and in the undifferentiated hyphae. Mutations resulting in loss-of-function in ATG22 expressed in gongylidia would explain the indefinite vacuolar swelling, as it would disable the efflux system and cause the cell to fill with cargo.

At the transcript level, a structurally modified and upregulated transcript carrying a domain associated with microtubule-related proteins (De Fine Licht et al. 2014) is believed to mediate such nuclear migration in association with motor protein complexes (Xiang et al. 1994; Yamamoto and Hiraoka 2003; Xiang 2018). This mechanism would resemble growth dynamics in most filamentous fungi, where nuclei are distributed evenly throughout the hyphal compartment and promote tip elongation by migrating apically (Plamann et al. 1994; Xiang 2018). Further supporting this hypothesis, alternative ramified branching patterns and intercalary bulbs occur when nuclei are occasionally aggregated in different regions of the filament and bulb. Finally, we propose that staphylae arise when these ramified branching patterns cause gongylidium cells to tangle in clusters (Fig. 4 D).

The migration of nuclei during gongylidium formation suggests a function for the extreme heterokary exhibited by the domesticated *L. gongylophorus* cultivar whose cells can contain up to 17 nuclei and up to 7 distinct haplotypes (Kooij et al. 2015). The next steps involve moving beyond distributions of nuclei in gongylidium cells, to tests of whether factors like nucleus-specific expression and nuclear dominance are linked to gongylidium formation. Such a mechanism has recently been observed in the production of edible reward structures produced by the heterokaryotic human-domesticated champignon fungus (*Agaricus bisporus*), where two distinct nuclear types exhibit differential expression in distinct tissues during mushroom formation (Gehrmann et al. 2018). The convergent existence of such molecular mechanisms in gongylidium formation would provide a further means of testing the hypothesis that these nutritional rewards are derived from cystidia (Mueller et al. 2001), which are modified hyphae found in the hymenium of several groups of basidiomycetes (Nobles 1965; Stalpers 1978). Autophagic induction of cystidia may have provided crucial pre-adaptations that were harnessed by natural selection to generate the unique gongylidium reward structures.

## CONCLUSIONS

This study details how autophagy drives the morphogenesis of the nutritional rewards produced by *Leucoagaricus gongylophorus* for their leafcutter ant farmers, providing a modern elaboration and theoretical framework for the initial 40-year old observations of Angeli-Papa and Eymé (1979, 1985). We used advanced imaging, in vitro experimentation, and transcriptomics approaches to show how autophagy mediates the production of nutritional rewards in a complex ectosymbiosis. In turn, we hypothesize that autophagy provides a mechanism for maintaining higher level homeostasis and thus raises fundamental questions about the levels of biological organization that can give rise to such resiliency. This autophagic mechanism, coupled with the observed bursting of unharvested staphylae, suggests that the autophagically derived nutrients are not recycled back into the fungus' metabolism. The farmer-only nutritional endpoint implies that gongylidia exemplify a true crop domestication. In combination, their distinctive morphology, ontogeny and physiology render gongylidia unique within the fungal kingdom.

### Supplementary Information

**Additional file 1: Figure S1.** Representative counts of gongylidia per staphyla in *L. gongylophorus* from colonies of different leafcutter ant species. **A** Staphylae from in-vitro Petri dish culture grown in absence of ant farmers. **B**–**E** Staphylae sampled directly from fungus garden in colonies having been actively farmed by ants. Yellow marks indicate individual gongylidia counted in the ImageJ program. Scale bars = 100 µm

**Additional file 2: Video S1.** Time lapse of staphyla stained with dextran-Alexa Fluor 647 under confocal microscope over 20 minutes. Red arrows point to gongylidia in which is possible to observe autophagic bodies trapped within the vacuole. Some of these vesicles are filled with dextran and appear more fluorescent than the vacuole lumen, others are filled with non-fluorescent material and appear darker than the vacuole.

**Additional file 3: Figure S2.** Mycelial growth distribution (area per treatment). The mycelial growth showed significant differences between 3-MA and all other treatments (PDA:3-MA, *p*_adj_ = 0.004; RAP:3-MA, *p*_adj_ = 0.015; CQ:3-MA, *p*_adj_ = 0.001), but no other significant pairwise comparisons. Horizontal bars indicate the distribution means.

**Additional file 4: Figure S3.** Autophagy metabolic pathway map displaying gene expression in *L. gongylophorus*. Genes highlighted in colours have upregulated transcripts in staphylae (yellow), mycelia (red) or both tissues (blue). The steps of the pathway are illustrated on the right showing where the products of these genes act during autophagy. The map was adapted from the KEGG website (www.kegg.jp).

## Data Availability

The sample Ac2012-1 is registered in NCBI BioSample with accession SAMN30820548 and the related RNA-seq data produced in this study is publicly accessible on the NCBI Sequence Read Archive (SRA) with accession numbers ranging from SRR21540775 to SRR21540784. Additionally, the transcriptome assembly, DET statistics and DET nucleotide and amino acid sequences are available at https://doi.org/10.17894/ucph.e6f0438f-e33d-4043-98aa-07de4d9fa9e3 (Leal-Dutra and Shik 2023).

## Abbreviations

TEM: Transmission electron microscopy
SEM: Scanning electron microscopy
RH: Relative humidity
PDA: Potato dextrose agar
PBS: Phosphate buffered saline
TOR: Target of rapamycin
RAP: Rapamycin
CQ: Chloroquine
3-MA: 3-Methyladenine
DET: Differentially expressed transcript

## Declarations

### Adherence to national and international regulations

The authors confirm that this manuscript respects the Nagoya Protocol to the Convention on Biological Diversity.
