# Supplementary material for: Evidence that the domesticated fungus Leucoagaricus gongylophorus recycles its cytoplasmic contents as nutritional rewards to feed its leafcutter ant farmers
Source: IMA Fungus. 2023 Sep 15;14:19. doi: 10.1186/s43008-023-00126-5 (PMC10503033; doi:10.1186/s43008-023-00126-5)
Supplement: Supplementary file 1 — Additional file 1: Figure S1. Representative counts of gongylidia per staphyla in L. gongylophorus from colonies of different leafcutter ant species. A Staphylae from in-vitro Petri dish culture grown in absence of ant farmers. B–E Staphylae sampled directly from fungus garden in colonies having been actively farmed by ants. Yellow marks indicate individual gongylidia counted in the ImageJ program. Scale bars = 100 µm [file 43008_2023_126_MOESM1_ESM.pdf]

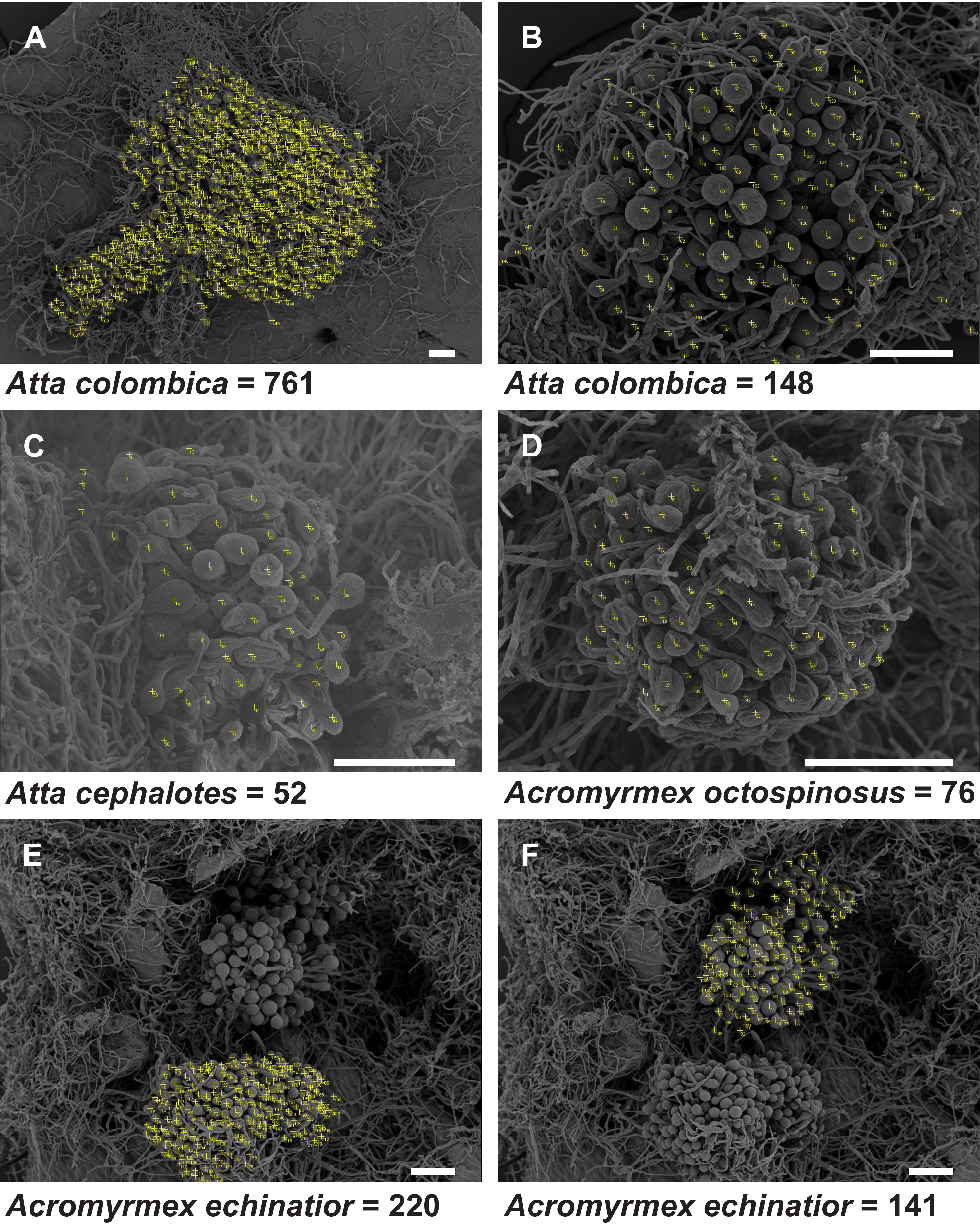
A
Atta colombica = 761
B
Atta colombica = 148
C
Atta cephalotes = 52
D
Acromyrmex octospinosus = 76
E
Acromyrmex echinatior = 220
F
Acromyrmex echinatior = 141
