# Supplementary figures and images for: Evidence that the domesticated fungus Leucoagaricus gongylophorus recycles its cytoplasmic contents as nutritional rewards to feed its leafcutter ant farmers
Source: IMA Fungus. 2023 Sep 15;14:19. doi: 10.1186/s43008-023-00126-5 (PMC10503033; doi:10.1186/s43008-023-00126-5)

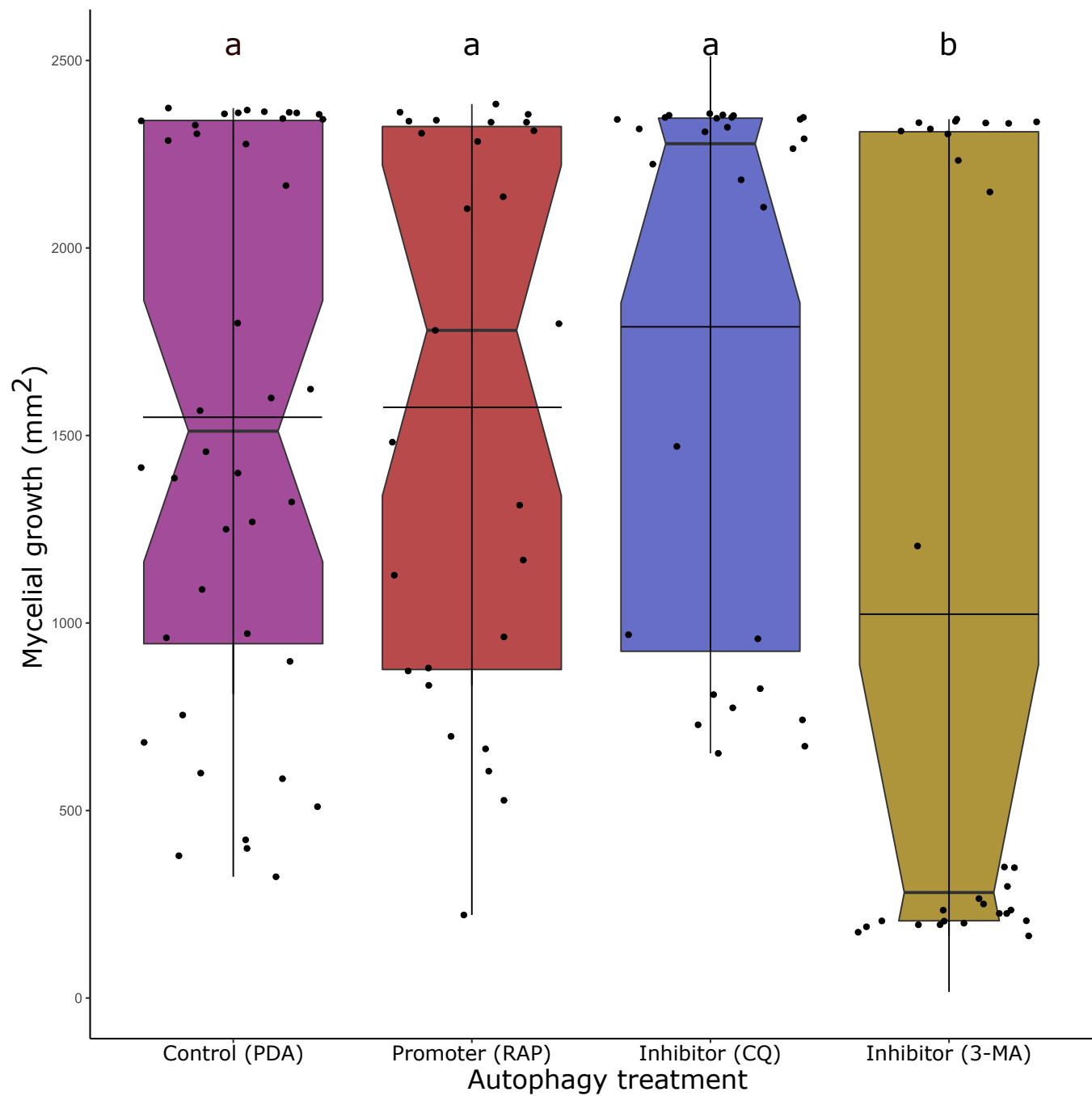

Supplement: Supplementary file 3 — Additional file 3: Figure S2. Mycelial growth distribution (area per treatment). The mycelial growth showed significant differences between 3-MA and all other treatments (PDA:3-MA, padj = 0.004; RAP:3-MA, padj = 0.015; CQ:3-MA, padj = 0.001), but no other significant pairwise comparisons. Horizontal bars indicate the distribution means. [file 43008_2023_126_MOESM3_ESM.pdf]

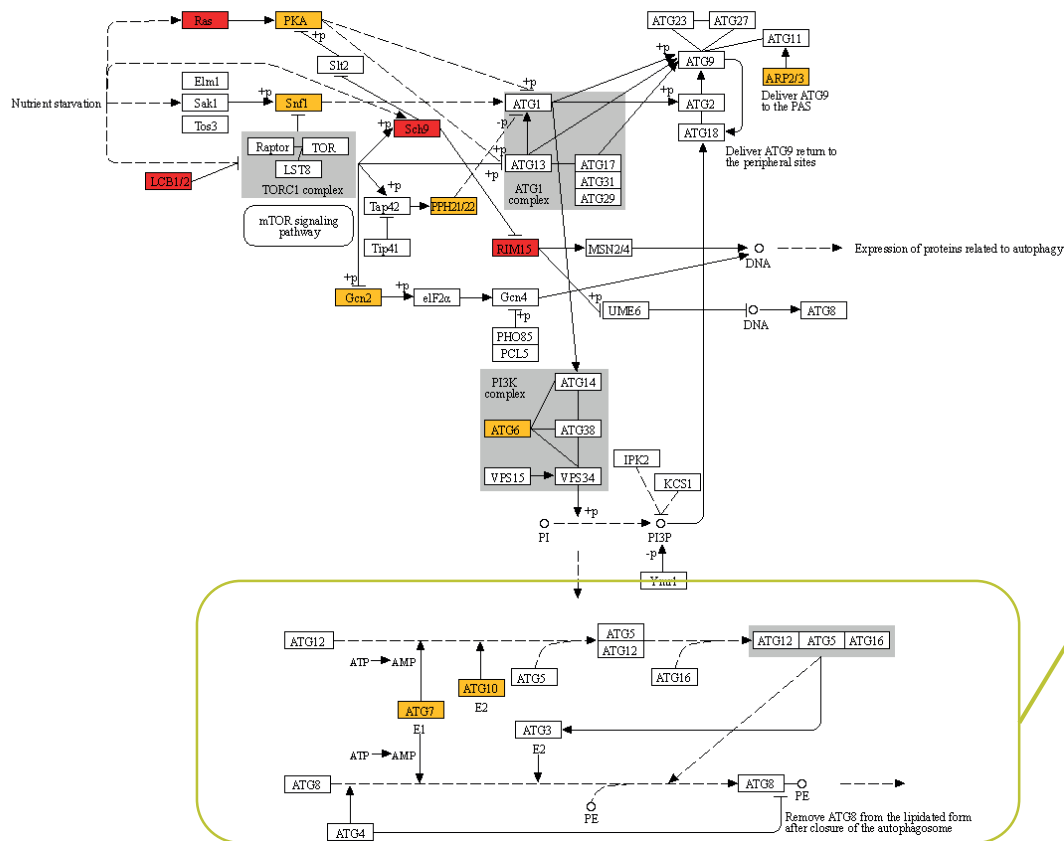

- Upregulated in staphyiae
- Upregulated in mycelium
- Upregulated in both

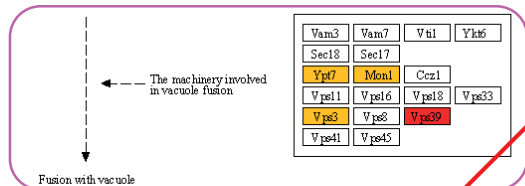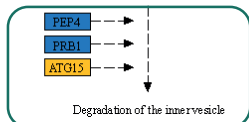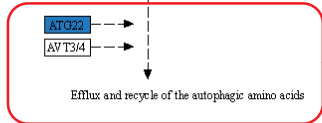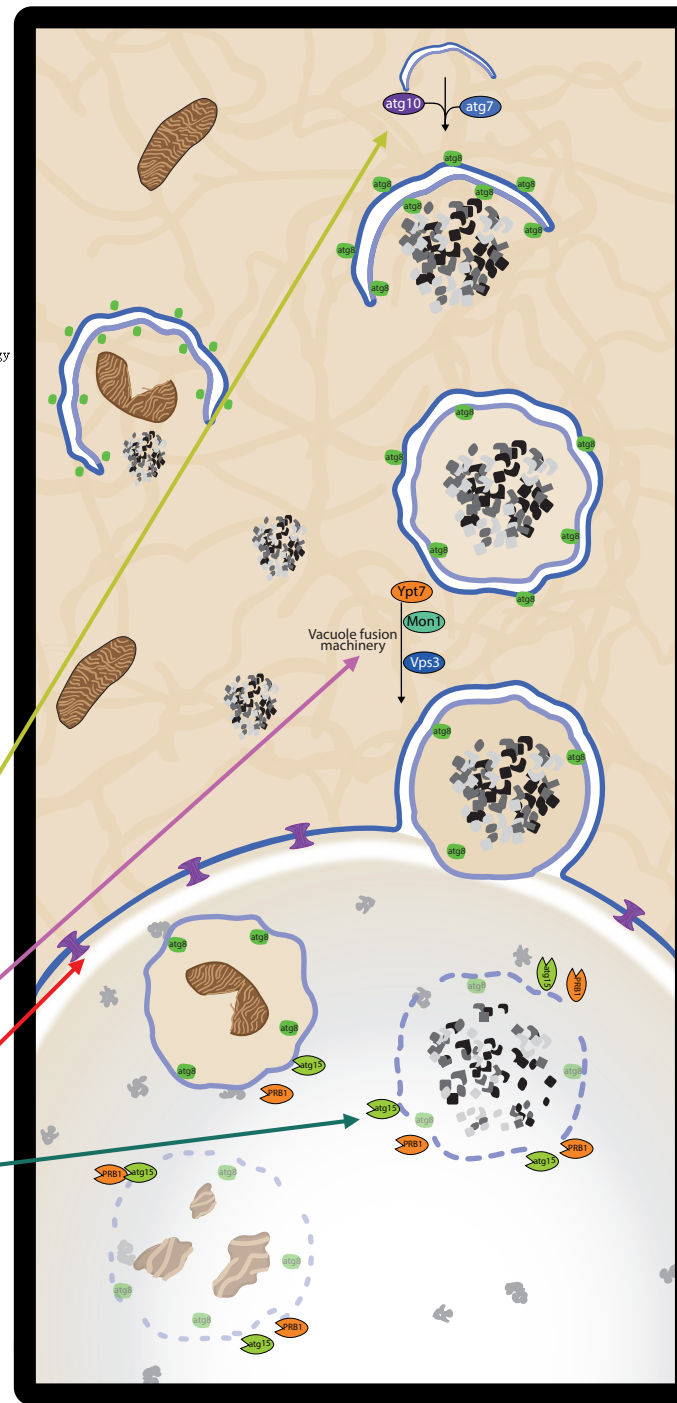

Supplement: Supplementary file 4 — Additional file 4: Figure S3. Autophagy metabolic pathway map displaying gene expression in L. gongylophorus. Genes highlighted in colours have upregulated transcripts in staphylae (yellow), mycelia (red) or both tissues (blue). The steps of the pathway are illustrated on the right showing where the products of these genes act during autophagy. The map was adapted from the KEGG website (www.kegg.jp). [file 43008_2023_126_MOESM4_ESM.pdf]
